# Solitary Granular Cell Tumor of Cecum: A Case Report

**DOI:** 10.5402/2011/943804

**Published:** 2010-11-04

**Authors:** Kaoutar Znati, Taoufiq Harmouch, Amal Benlemlih, Hinde Elfatemi, Laila Chbani, Afaf Amarti

**Affiliations:** Department of Pathology, Hassan II University Hospital, Fez, Morocco

## Abstract

Granular cell tumor (GCT) was first described by Abrikossof in 1926. This tumor is a benign neoplasm of unclear histogenesis that is generally believed to be of nerve sheath origin. GCT is not common and most often affects the tongue, skin, and soft tissue, although it may occur anywhere in the body. Gastrointestinal tract involvement, and especially that of the colon, is very rare. This usually benign tumor appears as a submucosal nodule, measuring less than 2 cm in diameter and is often found incidentally during colorectal examinations. We describe the case of a 27-year-old man with a GCT in the cecum that was detected after a screening colonoscopy. Endoscopic examination revealed a yellowish submucosal tumor, 0.7 cm in diameter. An endoscopic mucosal polypectomy was done for histological confirmation and treatment.

## 1. Introduction

Granular cell tumor (GCT) is a benign tumor with unknown histogenesis that is characterized by large granular eosinophilic cells [1]. GCT was first described by Abrikossoff in 1926, [2] as a muscle tumor, and several cases have been reported since then, but gastrointestinal tract involvement, and especially that of the colon, is very rare [3, 4]. A few cases of GCT have been reported in the cecum.

Instead, these tumors tend to be found incidentally during colonoscopic examinations performed for other reasons, as they are frequently asymptomatic. Endoscopically, a GCT usually appears as a small submucosal nodule, measuring less than 2 cm in diameter [4–7]. In the present paper, we describe a rare case involving a 27-year-old man diagnosed with a GCT arising in the cecum. The tumor was resected by endoscopic mucosal resection (EMR) for histological confirmation and treatment.

## 2. Case Report

**Figure 1:**
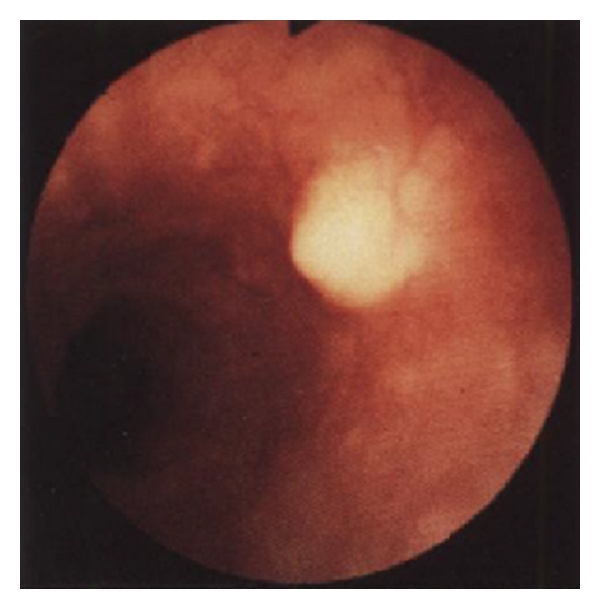
Endoscopic photograph demonstrating a yellowish submucosal tumor in the cecum.

A 27-year-old man was admitted to the Gastrointestinal Department of Hassan II University Hospital with abdominal pain and diarrhea. Colonoscopy revealed a polypoid mass measuring approximately 0.7 cm in diameter (Figure 1). An endoscopic mucosal polypectomy was done for histological confirmation and treatment.

**Figure 2:**
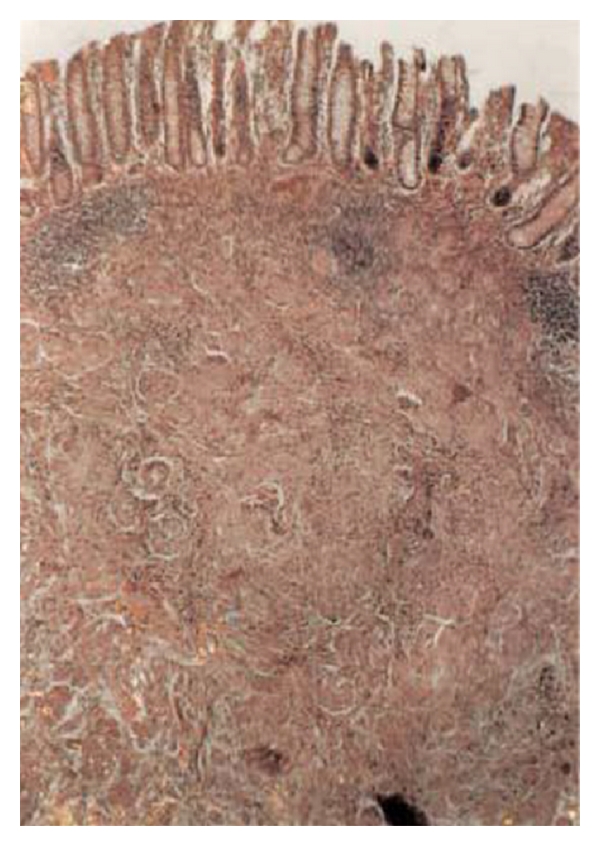
Low magnification view of submucosal tumor arranged in nodules and sheets (H&E × 4).

**Figure 3:**
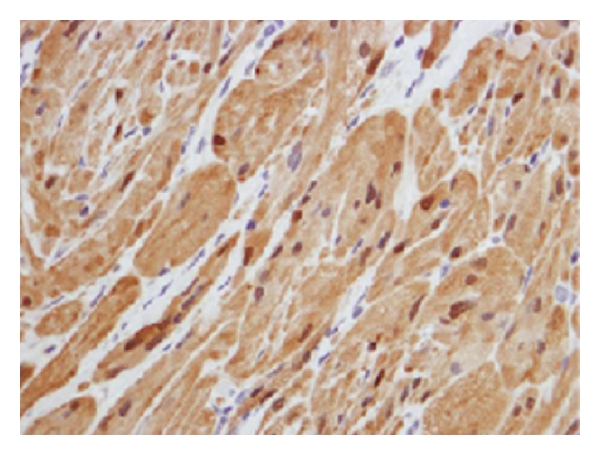
Histological findings of the tumor showing positive immunoreaction for S-100 protein (immunohistochemical  stain × 400).

In the cecum, a submucosal tumor was identified that was composed of solid masses of plump histiocyte-like cells with abundant granular eosinophilic cytoplasm with centrally located vesicular or dark pyknotic nuclei. In some areas, a slightly nodular architecture was identified. These nodules or sheets were surrounded by variable stroma (Figure 2). The granules observed in the cell cytoplasm stained positive with periodic acid-Schiff (PAS). Immunohistochemical analysis showed that the tumor cells expressed S-100 protein as well (Figure 3). During a 3-year followup, he has been well without disease recurrence.

## 3. Discussion

GCT is a rare tumor that usually appears as a solitary, small, nodular growth, and it follows a benign course. While GCTs may occur at any site of the body, they are most frequently detected in the oral cavity, skin, and subcutaneous tissue [8]. In the gastrointestinal tract, where GCTs are uncommon, the esophagus is the most frequent site, followed by the colon and stomach [7]. Colorectal GCTs may be located anywhere between in the colon. However, the most common locations for colorectal GCTs stated in the literature including approximately 130 cases of colorectal GCTs are the rectum and cecum [9]. The tumors usually present as solitary submucosal lesions, but a multifocal disease limited to the gastrointestinal tract or involving extragastrointestinal sites has been described, representing 10% to 20% of all GCTs [6, 7].

Most GCTs of the gastrointestinal tract are submucosal and thus are covered by normal mucosa [4]. GCTs are generally not large, being mostly between 1 and 2 cm in diameter; the largest reported lesion was 4 cm [8]. Therefore, these tumors are generally found incidentally. Endoscopy demonstrates a yellowish, submucosal tumor that is usually without mucosal ulceration and has a slightly rough surface [8, 10]. Recently, endoscopic ultrasonography (EUS) has been used more frequently to determine the depth of tumor invasion in the gastrointestinal wall, and it is useful for evaluating gastrointestinal tract submucosal tumors [11]. However, EUS cannot sufficiently distinguish a benign submucosal tumor from other tumors such as malignant neoplasia [12].

The final diagnosis of GCT depends on pathological findings. These tumors display three main architectural patterns, including small and well-circumscribed nodules, larger and poorly circumscribed lesions, and an impressive infiltrative pattern with remote satellite nodules. Neoplastic cells are plump histiocytelike, and bland looking with abundant granular eosinophilic cytoplasm containing acidophilic, PAS-positive, diastase-resistant granules; small, uniform nuclei, in which mitotic figures are absent; neural markers, including S-100 protein or NSE, expressed uniformly [13, 14].

The histogenesis of GCT has remained enigmatic in spite of a vast number of immunohistochemical and ultrastructural studies. Positivity for S-100 and nestin, also seen in gastrointestinal stromal tumors and schwannomas, suggests Schwann cell or Cajal cell derivation [14]. 

Neural origin or differentiation, in particular of the Schwann cell type, is currently in favor. However, recent findings have cast doubt on the neural origin of these tumors. Vered et al. [15] have suggested that immunoreactivity of the granular cells to a broad panel of antibodies including S-100 protein, CD68, vimentin, calretinin, NKI/C3, protein gene product 9.5, nerve growth factor receptor, and inhibin-A that characterize different tissues does not confirm any particular cell type for the histogenesis of GCT. They suggested that the lesions may be metabolic or reactive in nature and nonneoplastic. 

Malignant GCTs have been described but are extremely uncommon, representing 1% to 2% of all GCTs [16]. Only a few case reports have been published on gastrointestinal tract involvement; however, no colonic examples were recorded. Clinically, malignancy is suggested by GCTs rapid growth, size greater than 4 cm, and invasion of the adjacent organ. Despite the histologic similarity observed in benign and malignant GCTs, Fanburg-Smith et al. [17] proposed microscopic criteria to classify and predict the malignant potential of GCTs. These include necrosis, vesicular nuclei with large nucleoli, brisk mitotic activity (>2 per 10 high power fields at 200_magnification), high nuclear to cytoplasmic ratio, nuclear pleomorphism, and spindling. Neoplasms meeting at least 3 or more of these criteria were classified as histologically malignant; if 1 or 2 criteria are identified, then the tumors are classified as atypical, and those that displayed only focal nuclear pleomorphism but fulfilled none of the other criteria were classified as benign. Malignant potential has not been reported with colonic GCTs. Thus, patients are managed conservatively, with polypectomy for tumors less than 4 cm and segmental colectomy for larger masses. Endoscopic mucosal resection using a transparent cap has also been advocated. Its ease of use in submucosal dissection allows for clear margins, while minimizing the risk of perforation [18]. 

Although endoscopic resections of colorectal GCTs are curative in most cases, local recurrence has also been reported [8]. Evaluation of lateral and deep margins is needed to prevent recurrence.

Follow-up colonoscopic examination is necessary when the tumors are multiple or a risk of malignancy exists. In addition, endoscopic examination should include a baseline gastroscopy to exclude the presence of GCTs in the esophagus and the stomach, which are the other sites in the gastrointestinal tract where GCTs can be found.
